# Supplementary material for: Impact of varying accelerometer epoch length on physical activity patterns in adults: Considerations for public health
Source: PLoS One. 2024 Dec 31;19(12):e0316176. doi: 10.1371/journal.pone.0316176 (PMC11687789; doi:10.1371/journal.pone.0316176)
Supplement: S1 File — (DOCX) [file pone.0316176.s001.docx]

| Epoch length | Wear Time | SB | LPA | MPA | VPA | MVPA |
| --- | --- | --- | --- | --- | --- | --- |
| 1-sec | 881.4 ± 95.5 | 656.1 ± 98.4 | 126.5 ± 39.8 | 86.1 ± 36 | 12.7 ± 19.2 | 98.8 ± 45 |
| 5-sec | 881.2 ± 95.7 | 592.8 ± 101.8 | 203.7 ± 61.8 | 77.1 ± 36.2 | 7.6 ± 17 | 84.7 ± 45.2 |
| 10-sec | 881.3 ± 95.7 | 560.3 ± 104.3 | 245.3 ± 73.1 | 69.7 ± 36.4 | 5.9 ± 15.8 | 75.7 ± 45.3 |
| 15-sec | 881.3 ± 95.7 | 540.3 ± 105.7 | 271.7 ± 80 | 64 ± 36.4 | 5.3 ± 14.9 | 69.3 ± 45.1 |
| 30-sec | 881.5 ± 95.7 | 507.2 ± 108 | 315.8 ± 90.4 | 54 ± 36.2 | 4.4 ± 13.5 | 58.4 ± 44.6 |
| 60-sec | 881.9 ± 95.6 | 474.9 ± 109.9 | 358.6 ± 99.4 | 44.7 ± 36 | 3.8 ± 11.9 | 48.5 ± 43.9 |
| *P* value* | < 0.001 | < 0.001 | < 0.001 | < 0.001 | < 0.001 | < 0.001 |

**S1 Table.** Daily wear time and time spent in each physical activity intensity using different epoch lengths among male participants (n=80)

Abbreviations: SB: sedentary behaviour, LPA: light physical activity; MPA: moderate physical activity; VPA: vigorous physical activity; MVPA: moderate to vigorous physical activity

All values are expressed in mean minutes ± SD

*Repeated measures ANOVA.

**S2 Table.** Daily wear time and time spent in each physical activity intensity using different epoch lengths among female participants (n=101)

| Epoch length | Wear Time | SB | LPA | MPA | VPA | MVPA |
| --- | --- | --- | --- | --- | --- | --- |
| 1-sec | 874.4 ± 92.3 | 642.2 ± 101.3 | 139.3 ± 40.7 | 83.8 ± 36.3 | 9.1 ± 9.8 | 92.9 ± 40.6 |
| 5-sec | 873.1 ± 90.3 | 573.2 ± 106.4 | 222 ± 59.4 | 73.9 ± 37.7 | 4 ± 6.6 | 77.9 ± 40 |
| 10-sec | 873.2 ± 90.3 | 539.8 ± 110.1 | 264.7 ± 68.1 | 66.1 ± 37.6 | 2.6 ± 5.3 | 68.7 ± 39.5 |
| 15-sec | 873.4 ± 90.3 | 519.3 ± 112 | 292 ± 73.6 | 60.1 ± 37.1 | 2.1 ± 4.9 | 62.2 ± 38.8 |
| 30-sec | 873.6 ± 90.3 | 485.7 ± 115.1 | 337.1 ± 82.7 | 49.2 ± 35 | 1.6 ± 4.4 | 50.8 ± 36.5 |
| 60-sec | 873.9 ± 90.3 | 452.7 ± 117.4 | 380.1 ± 90.8 | 39.7 ± 32.5 | 1.3 ± 4 | 41 ± 33.8 |
| *P* value* | 0.58 | < 0.001 | < 0.001 | < 0.001 | < 0.001 | < 0.001 |

Abbreviations: SB: sedentary behaviour, LPA: light physical activity; MPA: moderate physical activity; VPA: vigorous physical activity; MVPA: moderate to vigorous physical activity

All values are expressed in mean minutes ± SD

*Repeated measures ANOVA.

**S3 Table.** Daily wear time and time spent in each physical activity intensity using different epoch lengths among participants aged 18-39 years (n=30)

| Epoch length | Wear Time | SB | LPA | MPA | VPA | MVPA |
| --- | --- | --- | --- | --- | --- | --- |
| 1-sec | 873.2 ± 94.3 | 655.5 ± 86.5 | 120.7 ± 31.1 | 79.4 ± 25 | 17.6 ± 11.7 | 97 ± 34 |
| 5-sec | 868.3 ± 87.6 | 585.4 ± 82.6 | 198.4 ± 47.2 | 74.8 ± 28.2 | 9.7 ± 9 | 84.4 ± 34.1 |
| 10-sec | 868.4 ± 87.6 | 551.6 ± 84.2 | 240.7 ± 55.3 | 69.5 ± 29.6 | 6.7 ± 7.7 | 76.2 ± 33.9 |
| 15-sec | 868.5 ± 87.6 | 530.4 ± 85.6 | 267.9 ± 60.4 | 64.6 ± 29.7 | 5.6 ± 7.3 | 70.2 ± 33.5 |
| 30-sec | 868.8 ± 87.5 | 495.3 ± 88 | 314.7 ± 69.8 | 54.6 ± 28.4 | 4.3 ± 6.6 | 58.9 ± 31.5 |
| 60-sec | 869.8 ± 87.2 | 460.5 ± 89.7 | 360.8 ± 77.8 | 44.9 ± 26.7 | 3.5 ± 6 | 48.4 ± 29.5 |
| *P* value* | 0.39 | < 0.001 | < 0.001 | < 0.001 | < 0.001 | < 0.001 |

Abbreviations: SB: sedentary behaviour, LPA: light physical activity; MPA: moderate physical activity; VPA: vigorous physical activity; MVPA: moderate to vigorous physical activity

All values are expressed in mean minutes ± SD

*Repeated measures ANOVA.

**S4 Table.** Daily wear time and time spent in each physical activity intensity using different epoch lengths among participants aged 40-54 years (n=60)

| Epoch length | Wear Time | SB | LPA | MPA | VPA | MVPA |
| --- | --- | --- | --- | --- | --- | --- |
| 1-sec | 886 ± 95.7 | 657.9 ± 109.3 | 130.8 ± 41.6 | 79.4 ± 30.4 | 17.9 ± 20.5 | 97.3 ± 45.4 |
| 5-sec | 886.1 ± 95.7 | 592.2 ± 113.3 | 210.6 ± 62.7 | 72.9 ± 33.5 | 10.4 ± 18.8 | 83.3 ± 46.7 |
| 10-sec | 886.2 ± 95.7 | 558.7 ± 115.5 | 253 ± 73 | 66.7 ± 35 | 7.8 ± 17.7 | 74.5 ± 47.3 |
| 15-sec | 886.6 ± 95.6 | 538.3 ± 116.9 | 280.2 ± 79.7 | 61.4 ± 35.9 | 6.8 ± 16.7 | 68.2 ± 47.7 |
| 30-sec | 886.9 ± 95.6 | 504.3 ± 118.9 | 325.4 ± 90.1 | 51.5 ± 36.4 | 5.7 ± 15.2 | 57.2 ± 47.5 |
| 60-sec | 887.5 ± 95.6 | 471.7 ± 121.1 | 368.6 ± 100.4 | 42.4 ± 37.1 | 4.8 ± 13.3 | 47.2 ± 47.1 |
| *P* value* | < 0.001 | < 0.001 | < 0.001 | < 0.001 | < 0.001 | < 0.001 |

Abbreviations: SB: sedentary behaviour, LPA: light physical activity; MPA: moderate physical activity; VPA: vigorous physical activity; MVPA: moderate to vigorous physical activity

All values are expressed in mean minutes ± SD

*Repeated measures ANOVA.

**S5 Table.** Daily wear time and time spent in each physical activity intensity using different epoch lengths among participants aged 55-74 years (n=91)

| Epoch length | Wear Time | SB | LPA | MPA | VPA | MVPA |
| --- | --- | --- | --- | --- | --- | --- |
| 1-sec | 873.3 ± 92.6 | 639.7 ± 98 | 139.9 ± 42.1 | 90.2 ± 41.7 | 3.6 ± 4.4 | 93.8 ± 43.5 |
| 5-sec | 873.3 ± 92.6 | 574 ± 105.5 | 221.2 ± 63.3 | 77 ± 41.6 | 1.1 ± 3.3 | 78.2 ± 42.2 |
| 10-sec | 873.3 ± 92.6 | 541.5 ± 109.8 | 263.2 ± 73.6 | 67.9 ± 40.7 | 0.8 ± 3.2 | 68.6 ± 41.2 |
| 15-sec | 873.3 ± 92.6 | 521.6 ± 112 | 289.9 ± 79.8 | 61.2 ± 39.5 | 0.6 ± 3.1 | 61.8 ± 40 |
| 30-sec | 873.3 ± 92.6 | 489.2 ± 115.4 | 333.5 ± 89.5 | 50 ± 37.2 | 0.5 ± 3 | 50.6 ± 37.8 |
| 60-sec | 873.3 ± 92.6 | 457.1 ± 117.8 | 375.1 ± 97.3 | 40.6 ± 34.5 | 0.5 ± 2.9 | 41.1 ± 35.1 |
| *P* value* | 0.42 | < 0.001 | < 0.001 | < 0.001 | < 0.001 | < 0.001 |

Abbreviations: SB: sedentary behaviour, LPA: light physical activity; MPA: moderate physical activity; VPA: vigorous physical activity; MVPA: moderate to vigorous physical activity

All values are expressed in mean minutes ± SD

*Repeated measures ANOVA.

**S6 Table.** Daily wear time and time spent in each physical activity intensity using different epoch lengths among participants with a BMI <25 (n=69)

| Epoch length | Wear Time | SB | LPA | MPA | VPA | MVPA |
| --- | --- | --- | --- | --- | --- | --- |
| 1-sec | 885 ± 79.8 | 649.9 ± 100.7 | 137.5 ± 40.8 | 85.8 ± 34.5 | 11.9 ± 18.7 | 97.6 ± 43.2 |
| 5-sec | 883.1 ± 76.5 | 581.7 ± 104.6 | 217.9 ± 59.6 | 77.2 ± 36.3 | 6.3 ± 16.4 | 83.5 ± 44.5 |
| 10-sec | 883.1 ± 76.5 | 548.7 ± 108 | 260 ± 67.9 | 69.7 ± 37.6 | 4.7 ± 15.0 | 74.4 ± 45.5 |
| 15-sec | 883.5 ± 76.5 | 528.5 ± 110.1 | 287.1 ± 73.6 | 63.9 ± 38.0 | 4 ± 14.0 | 67.9 ± 45.8 |
| 30-sec | 883.6 ± 76.5 | 494.6 ± 113.4 | 332.7 ± 82.5 | 53.0 ± 38.0 | 3.4 ± 12.6 | 56.4 ± 45.5 |
| 60-sec | 884 ± 76.5 | 460.6 ± 116 | 376.7 ± 89.2 | 43.8 ± 38.0 | 2.9 ± 10.8 | 46.7 ± 44.9 |
| *P* value* | 0.59 | < 0.001 | < 0.001 | < 0.001 | < 0.001 | < 0.001 |

Abbreviations: SB: sedentary behaviour, LPA: light physical activity; MPA: moderate physical activity; VPA: vigorous physical activity; MVPA: moderate to vigorous physical activity

All values are expressed in mean minutes ± SD

*Repeated measures ANOVA.

**S7 Table.** Daily wear time and time spent in each physical activity intensity using different epoch lengths among participants with a BMI ≥25 (n=112)

| Epoch length | Wear Time | SB | LPA | MPA | VPA | MVPA |
| --- | --- | --- | --- | --- | --- | --- |
| 1-sec | 872.9 ± 101.2 | 647.4 ± 100 | 131.3 ± 40.7 | 84.2 ± 37.2 | 9.9 ± 11.8 | 94.1 ± 42.3 |
| 5-sec | 872.8 ± 101.3 | 582.1 ± 105 | 211.5 ± 62 | 74.1 ± 37.4 | 5.2 ± 9.3 | 79.3 ± 41.2 |
| 10-sec | 872.8 ± 101.3 | 549 ± 108.1 | 253.7 ± 72.7 | 66.5 ± 36.9 | 3.7 ± 8.4 | 70.2 ± 40.1 |
| 15-sec | 872.9 ± 101.3 | 528.6 ± 109.6 | 280.6 ± 79.2 | 60.5 ± 36 | 3.2 ± 8.0 | 63.7 ± 39.1 |
| 30-sec | 873 ± 101.3 | 495.6 ± 112 | 324.6 ± 89.3 | 50.2 ± 34.1 | 2.5 ± 7.3 | 52.8 ± 37 |
| 60-sec | 873.4 ± 101.3 | 463.7 ± 113.9 | 366.8 ± 98.7 | 40.7 ± 31.6 | 2.1 ± 6.7 | 42.9 ± 34.4 |
| *P* value* | < 0.001 | < 0.001 | < 0.001 | < 0.001 | < 0.001 | < 0.001 |

Abbreviations: SB: sedentary behaviour, LPA: light physical activity; MPA: moderate physical activity; VPA: vigorous physical activity; MVPA: moderate to vigorous physical activity

All values are expressed in mean minutes ± SD

*Repeated measures ANOVA.
