# Supplementary material for: Impact of varying accelerometer epoch length on physical activity patterns in adults: Considerations for public health
Source: PLoS One. 2024 Dec 31;19(12):e0316176. doi: 10.1371/journal.pone.0316176 (PMC11687789; doi:10.1371/journal.pone.0316176)
Supplement: Attachment — Submitted filename: Response_to_reviewer_JV_CV.docx [file pone.0316176.s002.docx]

Dear Editor,

Thank you for these comments designed to improve our paper, “Impact of varying accelerometer epoch length on physical activity patterns in adults: considerations for public health” which we have addressed below. We greatly appreciate the time and effort put forth by reviewer to improve our paper. We hope our manuscript will now be suitable for publication in *Plos One*.

Sincerely,

Rayane Haddadj

**Major comments**

*1. Several cut-off points for the accelerometer-derived time spent in the different physical activity spectrum have been reported in the literature, and this is partly determined by several factors including the age distributions of the studied participants. The authors could help the reader by defining the specific cut-off thresholds for the different activity behaviours (sedentary behaviour and the different intensities of physical activity).*

**Thank you for this remark. We agree that providing the specific thresholds for the different activity behaviours would enhance the clarity of the manuscript. In response, we have added the exact accelerometer-derived thresholds used to classify sedentary behaviour and the various intensities of physical activity (light, moderate, and vigorous). These thresholds are now clearly defined, considering the age distribution of our study population, as supported by the relevant literature (lines 110 to 116).**

*2. Given the wide age range of the included participants, the authors could consider providing a brief justification of the 200 counts per minute (cpm) threshold for sedentary behaviour to help orient the reader.*

**We acknowledge the importance of justifying the 200 counts per minute (cpm) threshold for sedentary behaviour. To address this, we have included a relevant reference in the manuscript that supports the use of this threshold across different age groups (lines 112 and 114).**

*3. Another important factor which has been noted in the literature regarding activity behaviours (sedentary behaviour, and different intensities of physical activity) cut-off points selection for accelerometer raw data processing is the epoch length. The authors could clarify the cut-off thresholds applied for the various epoch lengths for the activity behaviours.*

**We now have clarified how the thresholds for sedentary behaviour and physical activity were adjusted according to the different epoch lengths used for accelerometer data processing (lines 100 and 101).**

*4. Did the authors consider checking or accounting for the potential influence of age distribution, gender and weight/BMI distribution on the estimated time spent in the activity behaviours for the various epoch lengths?*

**Thank you for this interesting suggestion. We have run additional analysis stratified on sex, age category and BMI status (Supplement files). We also commented those results in the text (line 149 and 150).**

**Minor comments**

*1. The authors mentioned in the abstract results that “Longer epochs led to reduced moderate and vigorous PA, increased light PA, and less sedentary time, affecting adherence to World Health Organization PA guidelines” (lines 23 – 25). But it is clear in the abstract if that was examined.*

**Thank you for this remark. To clarify this point, we have added a secondary objective in the abstract that states our examination of the effects of epoch length selection on computed prevalence of WHO physical activity guidelines (line 16 and 17).**

*2. The authors could be a little specific about the 13 participants excluded based on sociodemographic characteristics (…lines 83 – 84). Considering the nature of the analysis reported in the study, are the missing variables relevant for processing the accelerometer data?*

**We have now clarified that the 13 participants were excluded due to missing age data, which is critical for appropriately selecting the accelerometer thresholds (line 87, 88 and Figure 1).**

*3. There may probably be a typo in the description provided on …lines 113 – 115 regarding “qualitative”.*

**Thank you for pointing this out. We have corrected the typo by changing "qualitative" to "quantitative".**

*4. It may interest some readers if the pair-wise post hoc test results are also reported and interpreted as it may provide some relevant insights regarding epoch length selection.*

**We have added the pair-wise post hoc test results, in Table 2 and presented them in the results (lines 147 to 149) and discussion (line 184 to 186).**

*5. The authors could also consider discussing their observations within the context of cut-off points used for the data processing.*

**We have added a sentence in the limitations to highlight the context of cut-off points (line 241 and 242).**

*6. It may interest some readers if the authors also note in the discussion the potential influence of the body location where the participant wore the device on the estimation of the time spent in the activity behaviours.*

**Thank you for this interesting remark. We have added a comment on this topic in the limitations section (line 241and 242).**
